# Investigation of the 5q33.3 longevity locus and age-related phenotypes

**DOI:** 10.18632/aging.101156

**Published:** 2017-01-13

**Authors:** Marianne Nygaard, Mikael Thinggaard, Kaare Christensen, Lene Christiansen

**Affiliations:** ^1^ The Danish Aging Research Center, Epidemiology, Biostatistics and Biodemography, Department of Public Health, University of Southern Denmark, 5000 Odense C, Denmark; ^2^ Department of Clinical Genetics, Odense University Hospital, 5000 Odense C, Denmark; ^3^ Max Planck Odense Center on the Biodemography on Aging, 5000 Odense C, Denmark; ^4^ Department of Clinical Biochemistry and Pharmacology, Odense University Hospital, 5000 Odense C, Denmark

**Keywords:** 5q33.3, human longevity, age-related phenotypes, genetics

## Abstract

A large meta-analysis recently found the 5q33.3 locus to be associated with survival to ≥ 90 years and lower all-cause mortality, thus suggesting it as a third human longevity locus alongside *APOE* and *FOXO3A*. The 5q33.3 locus has previously been associated with blood pressure regulation and cardiovascular diseases in middle-aged individuals. However, part of the influence on mortality appears to be independent of cardiovascular phenotypes, and the role of the 5q33.3 locus in longevity and survival is therefore still partly unknown. We investigated the association between the longevity-associated variant rs2149954 on chromosome 5q33.3 and age-related phenotypes in two cohorts of 1,588 and 1,271 long-lived individuals (mean ages 93.1 and 95.9 years, respectively) as well as in 700 middle-aged and 677 elderly individuals (mean ages 52.5 and 78.7 years). Altogether, nominally significant associations between the rs2149954 minor allele and a decreased risk of heart attack and heart failure as well as increased physical functioning were found in the long-lived individuals. In the middle-aged and elderly individuals, rs2149954 minor allele carriers had a lower risk of hypertension. Our results thereby confirm a role of the 5q33.3 locus in cardiovascular health and, interestingly, they also suggest a role in physical functioning.

## INTRODUCTION

The search for major longevity genes in humans has so far had limited success and only the *APOE* and *FOXO3A* genes have been found to consistently associate with human longevity (e.g. [1–5]). Recently, however, a third longevity locus was proposed based on the results of a genome-wide association meta-analysis including 12,736 long-lived individuals ≥ 85 years and 76,268 younger controls < 65 years of European descent [6]. In this study, the single nucleotide polymorphism (SNP) rs2149954 on chromosome 5q33.3 was found to associate with survival to beyond 90 years of age (OR = 1.10, P = 1.74×10^−8^). This association has afterwards been confirmed in a genome-wide association study of exceptional longevity in Han Chinese centenarians (P = 0.02) [7]. Investigation of the effect of rs2149954 on prospective survival in the meta-analysis showed a significant association with lower all-cause mortality (HR = 0.95, P = 0.003) as well [6]. Further investigation of cause-specific mortality in a sub-group analysis revealed that the lower mortality seen in rs2149954 minor allele carriers was partly conferred by a decreased mortality risk for cardiovascular disease, primarily due to protection from stroke. However, a protective effect of the rs2149954 minor allele on mortality independent of cardiovascular disease was also found.

Previous studies in middle-aged individuals have revealed a significant association between the rs2149954 minor allele and a decreased risk for coronary artery disease, and lower diastolic and systolic blood pressure [6, 8, 9]. Also, two SNPs on chromosome 5q33.3 in high LD with rs2149954, rs9313772 and rs11953630, have been reported to be associated with blood pressure and hypertension [8, 10]. In individuals older than 75 years the association between rs2149954 and all-cause mortality was, however, not found to be influenced by blood pressure [6].

So, although there is an established connection between rs2149954 and different cardiovascular phenotypes, there also seems to be an effect of the variant in mechanisms other than those associated with cardiovascular disease and blood pressure regulation, at least in long-lived individuals. The role of the 5q33.3 locus in survival and longevity is therefore still partly unknown.

To further explore this, we investigated the influence of rs2149954 on age-related phenotypes previously shown to predict survival in the oldest-old [11, 12]: cognitive function (evaluated by a 5-item cognitive composite score and the Mini-Mental State Examination (MMSE)), physical function (evaluated by an activity of daily living (ADL) strength score, hand grip strength, gait speed, and chair stand), ADL disability, depression symptomatology, and self-rated health. In addition, self-reported diseases related to cancer and cardiovascular disease, which are among the leading causes of death in Denmark, were explored. The apparent age-dependent pleiotropy in the role of the 5q33.3 locus was addressed by analyzing long-lived as well as middle-aged and elderly individuals.

## RESULTS

Characteristics of the four study cohorts are summarized in Table 1, which also includes information on the rs2149954 minor allele frequency in each study cohort. The results of the association analyses between rs2149954 and the self-reported diseases and age-related phenotypes in the four study cohorts are shown in Table 2.

**Table 1:** Characteristics of the four study cohorts

	Middle-aged^1^	Elderly^2^	LLI-1^3^	LLI-2^4^
**N**	700	677	1,588	1,271
**Mean age (years)**	52.5	78.7	93.1	95.9
**Age range (years)**	45.9 – 59.0	73.0 – 95.5	92.2 – 93.8	94.7 – 100.9
**N Women (%)**	424 (60.6%)	448 (66.2%)	1,138 (71.7%)	928 (73.0%)
**Minor allele frequency, rs2149954**	0.3336	0.3597	0.3523	0.3533

1 Middle-aged refers to individuals from the Study of Middle-Aged Danish Twins (MADT).

2 Elderly refers to individuals from the Longitudinal Study of Aging Danish Twins (LSADT).

3 LLI-1: Long-lived individuals 1.

4 LLI-2: Long-lived individuals 2.

**Table 2:** Association between rs2149954 and the self-reported diseases and age-related phenotypes in the four study cohorts adopting an additive genetic model

	Middle-aged			Elderly			LLI-1			LLI-2		
**Self-reported Disease**	**N**	**OR**	**P**	**N**	**OR**	**P**	**N**	**OR**	**P**	**N**	**OR**	**P**
Cancer	700	0.85	0.684	677	0.73	0.082	1,579	1.02	0.905	1,265	1.03	0.830
Angina Pectoris	700	1.01	0.984	676	1.40	0.170	1,579	0.98	0.889	1,264	1.23	0.167
Heart Attack	700	1.01	0.994	677	0.89	0.612	1,581	0.74	**0.049**	1,266	1.13	0.404
Heart Failure	NA	NA	NA	676	0.83	0.378	1,577	0.78	**0.040**	1,263	1.03	0.897
Hypertension	700	0.65	**0.020**	676	0.69	**0.013**	1,567	0.97	0.779	1,261	0.94	0.492
Irregular Heart Rhythm	698	0.83	0.446	677	0.99	0.968	1,580	0.98	0.841	1,264	1.01	0.917
Other Heart Problems	700	0.79	0.691	675	1.34	0.252	1,579	0.96	0.789	1,259	0.97	0.801
Stroke	700	2.03	0.163	677	0.67	0.180	1,580	0.91	0.440	1,270	1.01	0.946
**Age-related Phenotype**	**N**	**OR**	**P**	**N**	**OR**	**P**	**N**	**OR**	**P**	**N**	**OR**	**P**
ADL Disability	NA	NA	NA	NA	NA	NA	1,586	0.92	0.259	1,266	1.08	0.327
ADL Strength	NA	NA	NA	676	1.27	0.066	1,571	1.18	**0.023**	1,254	0.94	0.440
Chair Stand	NA	NA	NA	NA	NA	NA	1,488	1.11	0.155	1,250	0.89	0.150
Chair Stand, Timed*	682	1.02	0.827	421	0.78	0.087	NA	NA	NA	NA	NA	NA
Depression	700	1.11	0.315	656	0.87	0.213	1,492	0.96	0.564	1,253	0.94	0.381
Gait Speed	NA	NA	NA	NA	NA	NA	1,314	1.04	0.617	1,041	1.05	0.616
MMSE	NA	NA	NA	657	0.96	0.701	1,522	1.04	0.598	1,246	0.96	0.627
Self-rated Health	700	0.84	0.105	661	1.12	0.294	1,526	1.02	0.819	1,265	1.06	0.416
**Age-related Phenotype**	**N**	**β**	**P**	**N**	**β**	**P**	**N**	**β**	**P**	**N**	**β**	**P**
Cognitive Composite Score	700	0.07	0.693	659	−0.07	0.727	1,518	0.02	0.877	1,244	−0.17	0.252
Grip Strength*	693	0.12	0.733	484	−0.03	0.931	1,424	0.04	0.858	1,091	−0.21	0.356

* In the elderly individuals these phenotypes were collected as part of the 1999 assessment of LSADT instead of the 1997 assessment. OR: Odds ratio. P: P-value obtained from logistic, linear, or ordinal logistic regression adjusted for age at assessment and gender. The P-values are not adjusted for multiple testing. P-values ≤ 0.05 are shown in bold. NA: Phenotype not available, or analysis not possible due to a low disease prevalence.

The investigated SNP was found to be in Hardy-Weinberg equilibrium in all study cohorts (P > 0.70, data not shown).

The primary focus of this study was to investigate the association between rs2149954 and age-related phenotypes, including self-reported diseases, in long-lived individuals. This was done in two study cohorts: long-lived individuals 1 (LLI-1) consisting of long-lived individuals from the 1905 Birth Cohort Study and long-lived individuals 2 (LLI-2) consisting of long-lived individuals from the 1910 and 1915 Birth Cohort Studies. When applying a stringent Bonferroni correction, no significant associations were found. However, in the LLI-1study cohort we found nominally significant associations between the minor allele of rs2149954 and a decreased risk of heart attack and heart failure. Also, minor allele carriers were found to have an increased ADL strength score, indicating a better physical functioning. In contrast, no nominally signi-ficant associations were found in the LLI-2 study cohort.

To also address the previously suggested age-related pleiotropy in the effect of the 5q33.3 locus, we analyzed the association between rs2149954 and available age-related phenotypes and self-reported diseases in middle-aged and elderly individuals as well. In the middle-aged individuals we found a nominally significant association between the rs2149954 minor allele dose and a lower risk for hypertension. A similar association was also seen in the elderly individuals, where we additionally found indications of an association between rs2149954 and a lower risk of cancer and increased physical performance represented by a higher ADL strength score and improved chair stand.

## DISCUSSION

In the present study we investigated the association between the longevity-related variant rs2149954 on chromosome 5q33.3 and age-related phenotypes, including selected self-reported diseases, in long-lived as well as in middle-aged and elderly individuals.

In LLI-1 we found a nominally significant association between rs2149954 and an increased ADL strength score. Also, we saw a nominally significant association with a decreased risk of heart attack and heart failure. These results support the previously suggested role of rs2149954 and the 5q33.3 locus in cardiovascular health [6, 8–10], and additionally they suggest a role of rs2149954 in physical functioning. It could be speculated that the influence of rs2149954 on physical functioning could somehow be mediated by the effect of rs2149954 on cardiovascular health. However, when adjusting for heart attack or heart failure in the analysis of the ADL strength score, the association remains the same, which could imply partly independent effects.

The nominally significant findings in LLI-1 could not be replicated in LLI-2, which may indicate that the findings in LLI-1 are chance findings. The lack of consistency could, however, also be a consequence of the difference of 2.8 years in mean age (93.1 years in LLI-1 vs. 95.9 years in LLI-2), which makes the selection pressure substantially higher in LLI-2 [13, 14]. Also, a recent study showed that nonagenarians from the 1915 Birth Cohort Study, who make up the majority of LLI-2, performed significantly better on cognitive tests and activity of daily living activities compared to nonagenarians from the 1905 Birth Cohort Study, who make up LLI-1 [15]. This together with the age difference could potentially dilute the genetic effect in LLI-2.

In the middle-aged and elderly individuals, we found a nominally significant association between the minor allele of rs2149954 and a lower risk of hypertension. This is supported by an analysis of the diastolic and systolic blood pressure measured in the middle-aged individuals at a later follow-up assessment (data not shown). Here we find that homozygous carriers of the rs2149954 minor allele have lower diastolic and systolic blood pressure, which is in line with the previously found association between rs2149954 and lower diastolic and systolic blood pressure in middle-aged individuals [6, 8].

Overall, our results support a role of rs2149954 in cardiovascular health, and we confirm the previously found association between rs2149954 and a lower risk of hypertension in middle-aged as well as in elderly individuals. The 5q33.3 locus thus appears to play a persistent role in cardiovascular health throughout the entire age-span investigated here, although we see a shift with age from a role in hypertension to a role in heart attack and heart failure. This shift is supported by a number of studies indicating that while high blood pressure is disadvantageous in midlife it appears to be advantageous at higher ages where it is associated with better physical and cognitive health and lower all-cause mortality [16–19]. This reversal of risk has been suggested to take place around the age of 75 to 85 years [20] and it is thus consistent with the age-related attenuation that we see for the association between rs2149954 and hypertension.

Our results also suggest a role for rs2149954 and the 5q33.3 locus in physical functioning. In a recent study, lower blood pressure in midlife was found to associate with better physical functioning in old age [21], which supports our results of a role for rs2149954 in hypertension in the middle-aged and elderly individuals and a role in physical functioning in the long-lived individuals.

In conclusion, our results point o a role of rs2149954 and 5q33.3 in cardiovascular health and physical functioning. Additional, preferably longitudinal and functional studies are needed to further improve the understanding of the role of this locus in longevity and survival.

## METHODS

### Study population

The individuals included in this study were middle-aged, elderly and long-lived participants from five different surveys conducted at the University of Southern Denmark.

The middle-aged individuals were drawn from the Study of Middle Aged Danish Twins (MADT). MADT was initiated in 1998 and includes 4,314 twins randomly chosen from the birth years 1931-1952 [22]. Surviving participants were revisited from 2008 to 2011[23]. Here, we included 700 twins randomly selected among twins born in 1940 or later. Only one twin from each twin pair was included.

The elderly individuals were drawn from the Longitudinal Study of Aging Danish Twins (LSADT). LSADT was initiated in 1995 and includes twins aged 70 years and older. Follow-up assessments were conducted every second year through 2005 [24]. The 677 twins included in the present study all participated in the 1997 assessment, where a total of 689 individuals provided a blood sample. Both twin pairs and singletons were included.

The long-lived individuals were drawn from three population-based nationwide surveys: the Danish 1905 Birth Cohort Study, the Danish 1910 Birth Cohort Study, and the Danish 1915 Birth Cohort Study (Rasmussen *et al*. 2016, submitted). Briefly, the Danish 1905 Birth Cohort Study was initiated in 1998, when participants were 92–93 years of age [25]. Follow-up assessments of participating survivors were carried out in 2000, 2003, and 2005. At intake there were 3,600 potential participants, of whom 2,262 agreed to take part in the survey. Among the 2,262 participants, 1,651 provided a biological sample, and 1,588 of these are included here. The Danish 1910 and 1915 Birth Cohort Studies include all Danes born in 1910 and 1915, and were initiated in 2010, when participants were 100 and 95 years of age, respectively [15]. In the 1910 Birth Cohort Study, a total of 400 individuals were invited to participate, which 273 individuals agreed to. Blood samples were retrieved from 176 individuals, of whom 175 are included here. In the 1915 Birth Cohort Study, 2,509 individuals were identified as eligible participants, and 1,584 individuals chose to participate. Blood samples were provided by 1,105 individuals, and 1,096 of these are included in this study.

Written informed consents were obtained from all participants, and all surveys, including collection of blood and use of survey information, were approved by the Regional Scientific Ethical Committees for Southern Denmark.

### Genotype data

DNA was extracted from dried blood spot cards using either the DNA Mini or Micro Kits (Qiagen, Hilden, Germany) or the Extract-N-Amp^TM^ Blood PCR Kit (Sigma-Aldrich, St. Louis, MO, USA), or from whole blood using a manual [26] or a semi-automatic (Autopure, Qiagen, Hilden, Germany) salting out method.

Genotyping of rs2149954 was performed using a predesigned TaqMan® SNP Genotyping Assay (Life Technologies, Carlsbad, CA, USA) following the manufacturer's instructions.

### Phenotype data

Data on the self-reported diseases and age-related phenotypes investigated in this study (see Supplementary Table 1) was collected as part of a comprehensive home-based interview focusing on health and lifestyle issues as well as objective assessments of cognitive and physical abilities.

Status on self-reported diseases was assessed by asking: ‘Did a doctor ever tell you that you have/had any of the following diseases?’ with the response categories ‘no’ or ‘has now or has had’. Cognitive function was assessed by the Mini-Mental State Examination (MMSE) and a cognitive composite score, which evaluates verbal fluency, forward and backward digit span, and immediate and delayed recall [27]. The MMSE score ranges from 0 to 30 and was here divided into four groups: severe impairment (MMSE 0-17), mild impairment (MMSE 18-22), normal (MMSE 23-27), and maximum (28-30). Physical function was assessed by an activity of daily living (ADL) strength score, chair stand, gait speed, and grip strength. The ADL strength score was calculated as the average of 11 individual items related to the ability to walk, run, climb stairs, and carry weights. Each item was scored from 1 to 4 with ‘1 = could not do’, ‘2 = could do with difficulty or an aid’, ‘3 = could do with fatigue’, and ‘4 = could do without fatigue’, and the average was subsequently categorized into three: ‘ADL strength < 2’, ‘ADL strength 2-<3’, and ‘ADL strength ≥ 3’. For chair stand different measures were used in the middle-aged and elderly individuals compared to the long-lived individuals. In MADT and LSADT, chair stand was measured as the time used (in seconds) to stand up from a chair five times in a row as quickly as possible and subsequently divided into quartiles. In the 1905, 1910, and 1915 Birth Cohort Studies, chair stand assessed the ability to stand up from a chair with the outcome categories ‘cannot’, ‘can, with use of arms’, and ‘can, without use of arms’. Gait speed was measured as a timed walk (in seconds) of a distance of 3 m and was here categorized into ‘cannot walk’, ‘speed ≤ 0.375 m/s’, and ‘speed > 0.375 m/s’. Grip strength was mea-sured using a handheld dynamometer (SMEDLEY's dynamometer, Scandidact, Kvistgaard, Denmark) and the maximum of three measurements with the strongest hand was used. The ADL disability score was based on a modified version of the Katz ADL index [28] and was computed from 8 questions relating to the five items bathing, dressing, toileting, transfer, and feeding. Here, the score was categorized into ‘not disabled = could do all 5 items’, ‘moderately disabled = could do 3 or 4 items’, or ‘disabled = could do maximum 2 items’. Depression symptomatology was assessed using an adaption of the depression section of the Cambridge Mental Disorders of the Elderly Examination [29]. Scores were grouped into four categories based on quartiles with higher scores reflecting a higher level of depression. Self-rated health was evaluated by asking: ‘How do you consider your health in general?’ with the five response categories ‘very poor’, ‘poor’, ‘acceptable’, ‘good’, and ‘excellent’.

### Statistical analyses

All statistical analyses were performed using the statistical software Stata (Stata version 13.1; Stata Corporation, College Station, TX, USA). Applying an additive genetic model with dose of minor allele (T) coded 0, 1, and 2, the association between rs2149954 and the self-reported diseases were assessed using logistic regression. Linear regression was used for the analysis of cognitive composite score and grip strength, and ordinal logistic regression was used for the analysis of ADL disability, ADL strength, chair stand, depression symptomatology, MMSE, and self-rated health. All analyses were adjusted for age at assessment and gender. In the analysis of LSADT, the within-pair dependency of twin pairs was taken into account by including the cluster option.

Given the a priori hypothesis of association between rs2149954 and longevity, blood pressure, and other cardiovascular phenotypes, and the correlation between the investigated phenotypes and diseases, a Bonferroni-corrected significance level of P ≤ 7.4× 10^−4^ (correcting for 17 age-related phenotypes and self-reported diseases in four study cohorts) is likely too stringent, and uncorrected P-values are thus reported.

Power calculations were performed in Quanto (version 1.2.4, http://biostats.usc.edu/Quanto.html) assuming an additive model, a rs2149954 minor allele frequency of 0.33 and a significance level of 0.05. For the continuous age-related phenotypes, cognitive composite score and grip strength, the calculations showed that effect sizes of 0.11 of a standard deviation (SD) or greater (corresponding to β-coefficients > 0.38 for cognitive composite score and β-coefficients > 0.74 for grip strength) could be detected with a power of at least 80%. For the self-reported diseases, the calculations showed that depending on disease prevalence odds ratios larger that 1.3-1.5 (or smaller than 0.67-0.77) could be detected with a power of at least 80%. These calculations are all based on LLI-1, which to some extend is our discovery cohort. For LLI-2, and the middle-aged and elderly individuals, the power to detect the above-mentioned effect sizes is, in most instances, slightly reduced due to smaller study cohort sizes and lower disease prevalence.

## SUPPLEMENTARY MATERIAL



## References

[1] Bao JM, Song XL, Hong YQ, Zhu HL, Li C, Zhang T, Chen W, Zhao SC, Chen Q. (2014). Association between FOXO3A gene polymorphisms and human longevity: a meta-analysis. Asian J Androl.

[2] Broer L, Buchman AS, Deelen J, Evans DS, Faul JD, Lunetta KL, Sebastiani P, Smith JA, Smith AV, Tanaka T, Yu L, Arnold AM, Aspelund T (2015). GWAS of longevity in CHARGE consortium confirms APOE and FOXO3 candidacy. J Gerontol A Biol Sci Med Sci.

[3] Christensen K, Johnson TE, Vaupel JW. (2006). The quest for genetic determinants of human longevity: challenges and insights. Nat Rev Genet.

[4] Schächter F, Faure-Delanef L, Guénot F, Rouger H, Froguel P, Lesueur-Ginot L, Cohen D. (1994). Genetic associations with human longevity at the APOE and ACE loci. Nat Genet.

[5] Willcox BJ, Donlon TA, He Q, Chen R, Grove JS, Yano K, Masaki KH, Willcox DC, Rodriguez B, Curb JD. (2008). FOXO3A genotype is strongly associated with human longevity. Proc Natl Acad Sci USA.

[6] Deelen J, Beekman M, Uh HW, Broer L, Ayers KL, Tan Q, Kamatani Y, Bennet AM, Tamm R, Trompet S, Guðbjartsson DF, Flachsbart F, Rose G (2014). Genome-wide association meta-analysis of human longevity identifies a novel locus conferring survival beyond 90 years of age. Hum Mol Genet.

[7] Zeng Y, Nie C, Min J, Liu X, Li M, Chen H, Xu H, Wang M, Ni T, Li Y, Yan H, Zhang JP, Song C (2016). Novel loci and pathways significantly associated with longevity. Sci Rep.

[8] Ehret GB, Munroe PB, Rice KM, Bochud M, Johnson AD, Chasman DI, Smith AV, Tobin MD, Verwoert GC, Hwang SJ, Pihur V, Vollenweider P, O'Reilly PF, CHARGE-HF consortium (2011). Genetic variants in novel pathways influence blood pressure and cardiovascular disease risk. Nature.

[9] Schunkert H, König IR, Kathiresan S, Reilly MP, Assimes TL, Holm H, Preuss M, Stewart AF, Barbalic M, Gieger C, Absher D, Aherrahrou Z, Allayee H, Cardiogenics, and CARDIoGRAM Consortium (2011). Large-scale association analysis identifies 13 new susceptibility loci for coronary artery disease. Nat Genet.

[10] Wain LV, Verwoert GC, O'Reilly PF, Shi G, Johnson T, Johnson AD, Bochud M, Rice KM, Henneman P, Smith AV, Ehret GB, Amin N, Larson MG, LifeLines Cohort Study, and EchoGen consortium, and Aorta-Gen Consortium, and CHARGE Consortium Heart Failure Working Group, and KidneyGen consortium, and CKDGen consortium, and Cardiogenics consor-tium, and CardioGram (2011). Genome-wide association study identifies six new loci influencing pulse pressure and mean arterial pressure. Nat Genet.

[11] Nybo H, Petersen HC, Gaist D, Jeune B, Andersen K, McGue M, Vaupel JW, Christensen K. (2003). Predictors of mortality in 2,249 nonagenarians--the Danish 1905-Cohort Survey. J Am Geriatr Soc.

[12] Thinggaard M, McGue M, Jeune B, Osler M, Vaupel JW, Christensen K. (2016). Survival Prognosis in Very Old Adults. J Am Geriatr Soc.

[13] Garagnani P, Giuliani C, Pirazzini C, Olivieri F, Bacalini MG, Ostan R, Mari D, Passarino G, Monti D, Bonfigli AR, Boemi M, Ceriello A, Genovese S (2013). Centenarians as super-controls to assess the biological relevance of genetic risk factors for common age-related diseases: a proof of principle on type 2 diabetes. Aging (Albany NY).

[14] Stevenson M, Bae H, Schupf N, Andersen S, Zhang Q, Perls T, Sebastiani P. (2015). Burden of disease variants in participants of the Long Life Family Study. Aging (Albany NY).

[15] Christensen K, Thinggaard M, Oksuzyan A, Steenstrup T, Andersen-Ranberg K, Jeune B, McGue M, Vaupel JW. (2013). Physical and cognitive functioning of people older than 90 years: a comparison of two Danish cohorts born 10 years apart. Lancet.

[16] Molander L, Lövheim H, Norman T, Nordström P, Gustafson Y. (2008). Lower systolic blood pressure is associated with greater mortality in people aged 85 and older. J Am Geriatr Soc.

[17] Oates DJ, Berlowitz DR, Glickman ME, Silliman RA, Borzecki AM. (2007). Blood pressure and survival in the oldest old. J Am Geriatr Soc.

[18] Sabayan B, Oleksik AM, Maier AB, van Buchem MA, Poortvliet RK, de Ruijter W, Gussekloo J, de Craen AJ, Westendorp RG. (2012). High blood pressure and resilience to physical and cognitive decline in the oldest old: the Leiden 85-plus Study. J Am Geriatr Soc.

[19] Szewieczek J, Dulawa J, Francuz T, Legierska K, Hornik B, Włodarczyk-Sporek I, Janusz-Jenczeń M, Batko-Szwaczka A. (2015). Mildly elevated blood pressure is a marker for better health status in Polish centenarians. Age (Dordr).

[20] Blom JW, de Ruijter W, Witteman JC, Assendelft WJ, Breteler MM, Hofman A, Gussekloo J. (2013). Changing prediction of mortality by systolic blood pressure with increasing age: the Rotterdam study. Age (Dordr).

[21] Strandberg AY, Strandberg TE, Stenholm S, Salomaa VV, Pitkälä KH, Tilvis RS. (2014). Low midlife blood pressure, survival, comorbidity, and health-related quality of life in old age: the Helsinki Businessmen Study. J Hypertens.

[22] Gaist D, Bathum L, Skytthe A, Jensen TK, McGue M, Vaupel JW, Christensen K. (2000). Strength and anthropometric measures in identical and fraternal twins: no evidence of masculinization of females with male co-twins. Epidemiology.

[23] Skytthe A, Christiansen L, Kyvik KO, Bødker FL, Hvidberg L, Petersen I, Nielsen MM, Bingley P, Hjelmborg J, Tan Q, Holm NV, Vaupel JW, McGue M, Christensen K. (2013). The Danish Twin Registry: linking surveys, national registers, and biological information. Twin Res Hum Genet.

[24] Skytthe A, Kyvik K, Holm NV, Vaupel JW, Christensen K. (2002). The Danish Twin Registry: 127 birth cohorts of twins. Twin Res.

[25] Nybo H, Gaist D, Jeune B, Bathum L, McGue M, Vaupel JW, Christensen K. (2001). The Danish 1905 cohort: a genetic-epidemiological nationwide survey. J Aging Health.

[26] Miller SA, Dykes DD, Polesky HF. (1988). A simple salting out procedure for extracting DNA from human nucleated cells. Nucleic Acids Res.

[27] McGue M, Christensen K. (2001). The heritability of cognitive functioning in very old adults: evidence from Danish twins aged 75 years and older. Psychol Aging.

[28] Katz S, Downs TD, Cash HR, Grotz RC. (1970). Progress in development of the index of ADL. Gerontologist.

[29] Roth M, Tym E, Mountjoy CQ, Huppert FA, Hendrie H, Verma S, Goddard R (1986). CAMDEX. A standardised instrument for the diagnosis of mental disorder in the elderly with special reference to the early detection of dementia. Br J Psychiatry.

