# Supplementary material for: Investigation of the 5q33.3 longevity locus and age-related phenotypes
Source: Aging (Albany NY). 2017 Jan 13;9(1):247–53. doi: 10.18632/aging.101156 (PMC5310666; doi:10.18632/aging.101156)
Supplement: Supplementary file 1 [file aging-09-0247-s001.pdf]

SUPPLEMENTARY MATERIAL

Supplementary Table 1. Descriptives of self-reported diseases and age-related phenotypes investigated in the four study cohorts.

	Middle-aged[1]		Elderly[2]		LLI-1[3]		LLI-2[4]	
**Self-reported Disease**	**N**	**No. individuals (%)**	**N**	**No. individuals (%)**	**N**	**No. individuals (%)**	**N**	**No. individuals (%)**
Cancer	700		677		1,579		1,265	
No		685 (97.9%)		593 (87.6%)		1,462 (92.6%)		1,131 (89.4%)
Has now or has had		15 (2.1%)		84 (12.4%)		117 (7.4%)		134 (10.6%)
Angina Pectoris	700		676		1,579		1,264	
No		694 (99.1%)		634 (93.8%)		1,410 (89.3%)		1,166 (92.2%)
Has now or has had		6 (0.9%)		42 (6.2%)		169 (10.7%)		98 (7.8%)
Heart Attack	700		677		1,581		1,266	
No		697 (99.6%)		635 (93.8%)		1,470 (93.0%)		1,149 (90.8%)
Has now or has had		3 (0.4%)		42 (6.2%)		111 (7.0%)		117 (9.2%)
Heart Failure	700		676		1,577		1,263	
No		698 (99.7%)		633 (93.6%)		1,390 (88.1%)		1,210 (95.8%)
Has now or has had		2 (0.3%)		43 (6.4%)		187 (11.9%)		53 (4.2%)
Hypertension	700		676		1,567		1,261	
No		612 (87.4%)		493 (72.9%)		1,246 (79.5%)		836 (66.3%)
Has now or has had		88 (12.6%)		183 (27.1%)		321 (20.5%)		425 (33.7%)
Irregular Heart Rhythm	698		677		1,580		1,264	
No		654 (93.7%)		591 (87.3%)		1,265 (80.1%)		975 (77.1%)
Has now or has had		44 (6.3%)		86 (12.7%)		315 (19.9%)		289 (22.9%)
Other Heart Problems	700		675		1,579		1,259	
No		693 (99.0%)		636 (94.2%)		1,452 (92.0%)		1,131 (89.8%)
Has now or has had		7 (1.0%)		39 (5.8%)		127 (8.0%)		128 (10.2%)
Stroke	700		677		1,580		1,270	
No		692 (98.9%)		639 (94.4%)		1,425 (90.2%)		1,118 (88.0%)
Has now or has had		8 (1.1%)		38 (5.6%)		155 (9.8%)		152 (12.0%)
**Age-related Phenotype**	**N**	**No. individuals (%)**	**N**	**No. individuals (%)**	**N**	**No. individuals (%)**	**N**	**No. individuals (%)**
ADL Disability	NA		NA		1,586		1,266	
Not disabled		NA		NA		815 (51.4%)		613 (48.4%)
Moderately disabled		NA		NA		586 (36.9%)		491 (38.8%)
Disabled		NA		NA		185 (11.7%)		162 (12.8%)
ADL Strength	NA		676		1,571		1,254	
< 2		NA		77 (11.4%)		865 (55.1%)		588 (46.9%)
2-<3		NA		130 (19.2%)		583 (37.1%)		419 (33.4%)
≥ 3		NA		469 (69.4%)		123 (7.8%)		247 (19.7%)
Chair Stand	NA		NA		1,488		1,250	
Cannot		NA		NA		103 (6.9%)		125 (10.0%)
Can, with use of arms		NA		NA		572 (38.4%)		558 (44.6%)
Can, without use of arms		NA		NA		813 (54.7%)		567 (45.4%)
Chair Stand, Timed*	682		421		NA		NA	
1st quartile		171 (25.1%)		108 (25.7%)		NA		NA
2nd quartile		170 (24.9%)		125 (29.7%)		NA		NA
3rd quartile		171 (25.1%)		84 (19.9%)		NA		NA
4th quartile		170 (24.9%)		104 (24.7%)		NA		NA

Depression Symptomatology<br>1st quartile<br>2nd quartile<br>3rd quartile<br>4th quartile	700	 253 (36.2%)<br>133 (19.0%)<br>157 (22.4%)<br>157 (22.4%)	656	 206 (31.4%)<br>139 (21.2%)<br>175 (26.7%)<br>136 (20.7%)	1,492	 378 (25.4%)<br>436 (29.2%)<br>342 (22.9%)<br>336 (22.5%)	1,253	 324 (25.9%)<br>314 (25.0%)<br>343 (27.4%)<br>272 (21.7%)
Gait Speed<br>Cannot walk<br>Speed ≤ 0.375 m/s<br>Speed > 0.375 m/s	NA	 NA<br>NA<br>NA	NA	 NA<br>NA<br>NA	1,314	 151 (11.5%)<br>303 (23.1%)<br>860 (65.4%)	1,041	 186 (17.9%)<br>196 (18.8%)<br>659 (63.3%)
MMSE<br>Severe impairment (0-17)<br>Mild impairment (18-22)<br>Normal (23-27)<br>Maximum (28-30)	NA	 NA<br>NA<br>NA<br>NA	657	 26 (4.0%)<br>68 (10.4%)<br>290 (44.1%)<br>273 (41.5%)	1,522	 307 (20.2%)<br>389 (25.5%)<br>618 (40.6%)<br>208 (13.7%)	1,246	 218 (17.5%)<br>281 (22.6%)<br>469 (37.6%)<br>278 (22.3%)
Self-rated Health<br>Very poor<br>Poor<br>Acceptable<br>Good<br>Excellent	700	 4 (0.6%)<br>27 (3.9%)<br>84 (12.0%)<br>271 (38.7%)<br>314 (44.8%)	661	 11 (1.7%)<br>36 (5.4%)<br>148 (22.4%)<br>245 (37.1%)<br>221 (33.4%)	1,526	 26 (1.7%)<br>128 (8.4%)<br>499 (32.7%)<br>615 (40.3%)<br>258 (16.9%)	1,265	 21 (1.7%)<br>53 (4.2%)<br>314 (24.8%)<br>525 (41.5%)<br>352 (27.8%)
**Age-related Phenotype**	**N**	**Mean (SE)**	**N**	**Mean (SE)**	□	**Mean (SE)**	**N**	**Mean (SE)**
Cognitive Composite Score	700	5.61 (0.12)	659	0.98 (0.13)	1,518	0.21 (0.09)	1,244	0.47 (0.10)
Grip Strength*	693	37.80 (0.47)	484	24.31 (0.39)	1,424	16.28 (0.17)	1,091	15.93 (0.20)

[1] Middle-aged refers to individuals from the Study of Middle-Aged Danish Twins (MADT). [2] Elderly refers to individuals from the Longitudinal Study of Aging Danish Twins (LSADT). [3]LLI-1: Long-lived individuals 1. [4]LLI-2: Long-lived individuals 2. *In the elderly individuals these phenotypes were collected as part of the 1999 assessment of LSADT instead of the 1997 assessment. NA: Phenotype not available.
